# On the Anisotropic Mechanical Properties of Selective Laser-Melted Stainless Steel

**DOI:** 10.3390/ma10101136

**Published:** 2017-09-26

**Authors:** Leonhard Hitzler, Johann Hirsch, Burkhard Heine, Markus Merkel, Wayne Hall, Andreas Öchsner

**Affiliations:** 1 Griffith School of Engineering, Griffith University, Gold Coast Campus, Southport 4222, Australia; W.Hall@griffith.edu.au (W.H.); A.Oechsner@griffith.edu.au (A.Ö.); 2 Faculty of Mechanical Engineering and Material Science, Aalen University of Applied Sciences, 73430 Aalen, Germany; Hirsch-Johann@web.de (J.H.); Burkhard.Heine@hs-aalen.de (B.H.); Markus.Merkel@hs-aalen.de (M.M.)

**Keywords:** tensile strength, hardness, microstructure, grain morphology, epitaxial grain growth, scan strategy, directional dependencies

## Abstract

The thorough description of the peculiarities of additively manufactured (AM) structures represents a current challenge for aspiring freeform fabrication methods, such as selective laser melting (SLM). These methods have an immense advantage in the fast fabrication (no special tooling or moulds required) of components, geometrical flexibility in their design, and efficiency when only small quantities are required. However, designs demand precise knowledge of the material properties, which in the case of additively manufactured structures are anisotropic and, under certain circumstances, inhomogeneous in nature. Furthermore, these characteristics are highly dependent on the fabrication settings. In this study, the anisotropic tensile properties of selective laser-melted stainless steel (1.4404, 316L) are investigated: the Young’s modulus ranged from 148 to 227 GPa, the ultimate tensile strength from 512 to 699 MPa, and the breaking elongation ranged, respectively, from 12% to 43%. The results were compared to related studies in order to classify the influence of the fabrication settings. Furthermore, the influence of the chosen raw material was addressed by comparing deviations on the directional dependencies reasoned from differing microstructural developments during manufacture. Stainless steel was found to possess its maximum strength at a 45° layer versus loading offset, which is precisely where AlSi10Mg was previously reported to be at its weakest.

## 1. Introduction

Additive manufacturing (AM) methods, such as the selective laser melting (SLM), represent powerful freeform fabrication techniques which can fabricate direct deployable components without the necessity of special tooling, and are highly efficient when only small quantities are required [1,2,3]. Since full melting of the raw metal powder enables the generation of fully dense parts within a single production step, with mechanical properties exceeding the specifications of the conventional material, the fabrication of highly specialized components (like tools, moulds, ultra-lightweight components or medical implants) using AM is increasing [4,5,6,7]. One of the major challenges to date is the characterization and prediction of the properties of additively manufactured structures and their linkage with the selected fabrication settings [8]. The approach most utilized for describing the manufacturing process is through the energy input of the laser beam per unit volume, commonly referred to as the energy density [9].

Enery density WS=laser power (W)layer thickness (m)×hatch distance (m)×scan speed (ms)

Unfortunately, it appears that this convenient approach through characterization with a single number is not able to sufficiently express the entire complexity of powder-bed based AM processes, like SLM [10,11,12]. Thus, at this stage, a proper description of the manufacturing process still requires the listing of the individual irradiation parameters. Hu et al. [13] highlighted the importance of the scan speed and the layer thickness. In addition to the pure irradiation, information about the raw metal powder, mainly its size and distribution, is also of great importance and should not be neglected. Spierings et al. [14] pointed out the necessity of having both small and large powder particles: fine particles are easily molten and favour a good relative density and surface quality; whereas larger particles benefit ductility. On this note, the mechanical properties, such as hardness and tensile strength, greatly correspond to the relative density, which is without doubt the most utilized characteristic for evaluating the quality of fabricated components [10]. To illustrate its importance, the aeronautical industry introduced a minimum relative density of 99% as a standardized quality requirement [15].

The properties of AM-fabricated components are known to be anisotropic, for reasons to do with their layer-wise generation; and, in addition, inhomogeneous, with the latter being related to location-dependent alterations caused by prolonged dwell-times at elevated temperatures. It has been shown that inhomogeneities, which are caused through age-hardening (e.g., in aluminium–silicon alloys or steel that is age-hardened) can be overcome using a post-heat treatment [16,17]. Differing statements and conclusions about the inherent anisotropy were reported in former studies on stainless steel. For example, outcomes about the directional dependencies of the tensile strength differ widely. For the polar angle (the inclination to the layers), the findings range from the predominantly accepted formulation (the highest tensile strength and highest breaking elongation are found in a parallel layer to the loading direction scenario; and the lowest results, with an almost linear tendency, are found when the loading direction is in an in-built direction, i.e., perpendicular to the layers) to the following particular findings [4,18]: Sehrt and Witt [19] reported the opposite case, with the highest breaking elongation being obtained in the perpendicular loading scenario; Rehme and Emmelmann [20] stated that the lowest results for both the ultimate tensile strength and the breaking elongation occurred under a 75° angle to the layers, whereas the maximum values for both were examined under a 15° angle; Tolosa, et al. [21] found an increase in strength by increasing the inclination angle from 30° to 45°; whereas Guan et al. [22], on the other hand, reported a minimum strength occurring at a 45° inclination. In a similar way, differing findings were also reported for the in-plane dependencies. In general, the effects occurring in-plane are less pronounced; Sehrt and Witt [19] even described this influence as being negligible. However, there are various findings and it can be concluded that the in-plane dependencies are highly influenced by the chosen scan strategy, and thus, there is a need to consider them dependent on the individual settings [1]. Niendorf et al. [23] stated that, in addition to the irradiation settings, the dimensions of the structure also affected the mechanical strength and altered the microstructure. This finding was confirmed by Niendorf et al. [24], reporting noteworthy fluctuations of the mechanical properties and a strongly textured microstructure when high-energy laser systems (1000 W) are utilized. Rashid et al. [17] documented that the scan strategy impacted the martensite/austenite ratio in 1.4542, and even caused minor changes in the dimension of the component. Similar findings were made by Mahmoudi et al. [25] who, in addition, highlighted that additively manufactured components show, in general, better performance in compressive loading.

Another aspect to consider is the influence of the surface roughness on the material strength, with machined components yielding better results due to the removal of cracks and defects on, or close to, the surface [19,26]. Apart from conventional subtractive procedures, multiple contactless surface modification feasibility studies have been undertaken which all share the benefit of offering a similar geometric flexibility to AM. Alrbaey et al. [27] and Schanz et al. [28,29] investigated the surface laser-polishing of AM components and reported successful roughness reductions in the range from 80% to 92%. In addition to surface quality enhancement, the laser surface modification can also be utilized to adjust surface hardening, as shown by Martínez et al. [30]. AlMangour and Yang [31] showed that severe shot peening is another promising process that can greatly improve the mechanical properties of the surface. However, pure strength aside, AM provides the unique opportunity to directly incorporate additional features, such as textured surfaces to lower the friction, or bio-activated (open-porous) surface structures to enhance bone-ingrowth [32,33].

Besides its well-known geometric freedom and greatly enhanced yield strength, AM offers additional opportunities to further enhance material strength [34]. In recent studies the performance gain of 1.4404 through particle reinforcement was extensively investigated. Reinforcement through TiC particles was shown to be capable of almost doubling the hardness with respect to the un-reinforced material [35,36]; and reinforcement via TiB2 was capable of almost tripling hardness [37]. In addition to the remarkable gain in surface hardness, refined microstructural characteristics and a reduced wear rate were reported.

In this study, the anisotropic material properties of stainless steel were examined with destructive material tests, since the characterisation of the anisotropic material properties via non-destructive procedures was found to be inadequate [38]. Moreover, the findings of these material tests were compared with the results reported in literature in order to acquire a comprehensive overview of the inherent directional dependencies and their variation among various machinery and irradiation settings. Special consideration is given to the scan strategy settings and microstructural development throughout the process.

## 2. Methodology

### 2.1. Manufacturing Conditions

**Figure 1:**
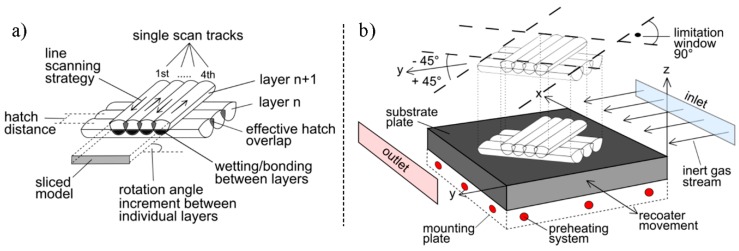
Schematic depiction of the SLM process; (**a**) the representation of the geometry via single scan tracks and layers; (**b**) the build environment; adapted from [8].

In this study, a SLM 280HL machine (SLM Solutions GmbH, Lübeck, Germany) equipped with a 400 W Yb-fibre-laser was utilized to manufacture the specimens. It features an available build space of 280 × 280 × 320 mm^3^ and includes a preheating system. The selected fabrication parameters are summarized in Table 1 and a brief explanation is provided in Figure 1. Low-carbon stainless steel type EN 1.4404, US 316L (also known as X2CrNiMo17-12-2) was chosen as the raw material and was supplied by SLM Solutions with the following properties: a mean particle diameter of 35.5 μm and an apparent powder density of 3.85 g/cm^3^ [39].

**Figure 2:**
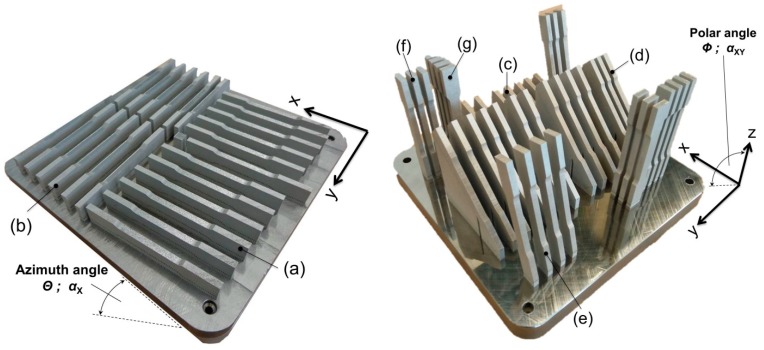
Tensile samples on the substrate plate, overview of the positioning and arrangement.

The tensile specimens were designed in accordance with the German standard DIN 50125:2009-07 [40] as flat specimens type E 5 × 10 × 40 and fabricated in seven distinct orientations (Table 2, Figure 2), subsequently referred to as configurations (a) to (g). The azimuth angle (*Θ*) describes the inclination of the sample to the *x*-axis and the polar angle (*Φ*) describes the inclination in relation to the xy-plane. The samples were fabricated with an oversize of 0.4 mm in width and thickness and milled to their final shape. On a side note, various techniques and alternatives for the preparation of tensile samples, to the chosen milling process, were investigated by Krahmer et al. [41]. Detailed studies on the effects of the positioning and inclination on the as-built surface roughness can be found elsewhere [39,42].

### 2.2. Material Testing

#### 2.2.1. Composition and Density

The material composition was determined with optical emission spectrometry (Q4 TASMAN, Bruker Corp., Billerica, MA, USA) on the machined samples. Based on the determined composition and the corresponding theoretical density, the relative density was obtained via the Archimedes method.

#### 2.2.2. Hardness

Surface hardness tests were systematically conducted on the clamping areas of the as-built and machined samples. Four indentations were evaluated on each sample. The hardness measurements were undertaken with a Reicherter KF hardness tester (Reicherter Georg GmbH Co Kg, Esslingen, Germany) in accordance to the DIN EN ISO 6507-2:2016 standard [43]. The testing force was set to 294.1 N and the hardness was obtained in HV30.

#### 2.2.3. Tensile Testing

**Figure 3:**
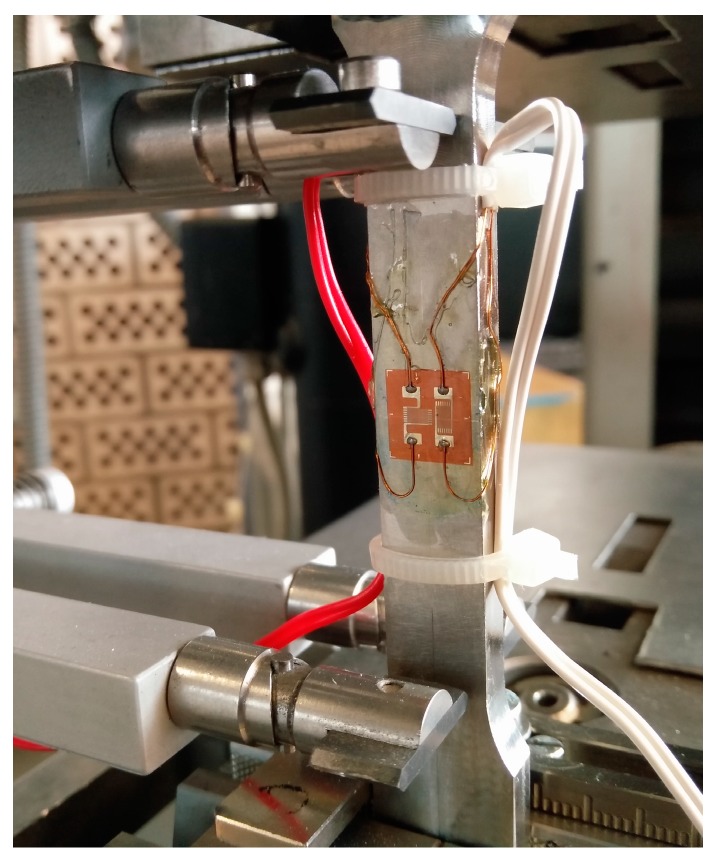
Tensile testing setup with extensometer and strain gauge.

For the destructive material tests, a tensile testing machine (Zwick/Roell, Ulm, Germany) with an in-built extensometer was utilized. The maximum load for this machine and the employed load cell was 100 kN, and the initial distance of the extensometer was set to 50 mm. The testing procedure was carried out in accordance with the German standard DIN EN ISO 6892-1:2016 [44] with a constant crosshead speed of 5 mm/min, and a total of 42 samples (6 samples per configuration) were tested. In addition, 3 out of 6 samples per configuration were equipped with an additional strain gauge (Type FCB-2-17-1L, Tokyo Sokki Kenkyujo Co., Ltd., Tokyo, Japan), comprising two individual measurement grids in a perpendicular arrangement to each other (each with a size of 1.5 × 2.5 mm^2^). The tensile setup is depicted in detail in Figure 3.

#### 2.2.4. Microstructure

For investigations of the microstructure, segments were taken from the tensile samples, which were embedded in a hot mounting resin. Various grinding and mechanical polishing steps were performed to expose the metallurgical structure. The visibility of the scan track pattern and the inherent grain structure was enhanced through a subsequent etching process. High-resolution images of the etched micro-sections were taken with an optical light microscope (Carl Zeiss Microscopy GmbH, Jena, Germany).

## 3. Results and Discussion

### 3.1. Density and Composition

The chemical composition of numerous samples of both batches was examined, and the averaged compositions are shown in Table 3. In short, the deviations among the two batches are negligible and are within the specifications of 1.4404. With the Archimedes method, a consistent relative density greater than 99% (machined condition) was determined.

### 3.2. Hardness

The hardness results were consistent in all directions, and the obtained results ranged from 223 to 234 HV for the machined condition and, respectively, from 235 to 245 HV in the as-built condition (Table 4), which is in perfect agreement with the documented values in the literature (Table 5). Throughout all the configurations, the surface hardness in the as-built condition exhibited an increased hardness of, on average, about 11 HV higher.

Unlike the previous investigation on AlSi10Mg, the hardness measurements did not exhibit noteworthy deviation along the specimens, indicating a homogeneous structure of the 1.4404 samples [16]. The reason for the inhomogeneities encountered in the AlSi10Mg samples was related to the alloy being age-hardened. Precipitation hardening occurred within the process, triggered by the dwell times at elevated temperatures in the build chamber. In the case of stainless steel, the difference between the temperature present in the build chamber compared to its melting temperature was greater, by far, than was the case for AlSi10Mg. Thus, for 1.4404 it can be concluded that the risks of inducing microstructural inhomogeneities due to dwell times are negligible at this point. Yadroitsev et al. [45] have shown that preheating temperatures above 500 °C result in the formation of satellites adjoined to the scan tracks and, in addition, the generation of unwanted splatters, that negatively affect the surrounding powder-bed. Given this upper limit for suitable preheating temperatures, coupled with the finding of Krakhmalev et al. [46] that the microstructural characteristics of 1.4404 stays stable up to 900 °C before significant grain-coarsening occurred, it can be concluded that inhomogeneities are unlikely to occur in 1.4404. It should, however, be noted that the correlated stress-relieving effects at higher temperatures lowered the hardness results [47]. Furthermore, one exception should be emphasised: in cases where the solidification morphology, i.e., columnar or equiaxed growth morphology, is crucial, the temperature field and, accordingly, the preheating temperature, is key for successful fabrication [48]. This is the case, for example, when single crystal structures are manufactured. However, for steel types capable of being age-hardened (such as the 1.4542 (17-4PH) stainless steel) the risk of inhomogeneous altering of the properties within the process cannot be ignored; AlMangour and Yang [49] pointed out that for additively manufactured 1.4542, an age-hardening with superimposed grain-coarsening occurred at 480 °C.

**Figure 4:**
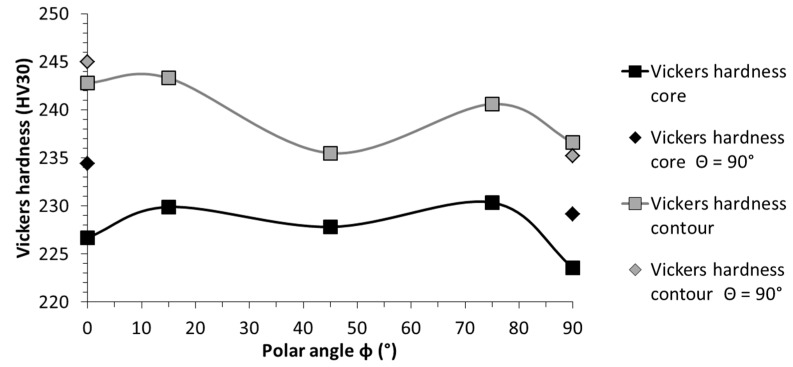
Orientation dependency of the surface hardness in both the as-build and machined condition.

One noteworthy and consistent relationship stood out in this study: comparing the configurations with identical polar angles, the configurations with a 90° azimuth angle revealed higher core hardness results (Figure 4). This finding will be addressed at a later point, together with the findings of the tensile test and micro-sections.

### 3.3. Tensile Strength

The samples throughout all configurations revealed arbitrary occurrences of failure along the gauge length, thus confirming the presence of a holistic homogeneous structure. Hence, the deviations subsequently discussed originated solely in the inherent directional dependencies, yet differentiations were necessary for both the polar and azimuth angles. The averaged results of the tensile test, as well as their according standard deviations, are presented in Table 6. These are drawn from six samples each, except for Poisson’s ratio, which is based on three measurements each. The latter will be investigated in more detail in a separate publication, since the encountered findings are scattered throughout the entire possible range of Poisson’s ratio (i.e., between 0 and 0.5 [55]), with one exception even being outside this range, which necessitates consideration of theories applicable to porous and composite material that can exhibit Poisson’s ratios greater than 0.5 [56].

As with the hardness evaluation, the samples with an azimuth angle of 90° stood out. Considering the in-plane oriented configurations (i.e., (a) and (b)), the Young’s modulus differed by more than 30%, whereas the deviations in yield strength and UTS were marginal. Interestingly, this considerable dependency of Young’s modulus on the azimuth angle was only present for the samples with a polar angle of zero degrees. This finding contradicts Sehrt and Witt [19], who reported that the in-plane orientation can be neglected. However, this simplification was qualified; Sehrt [1] added that the in-plane tendencies correspond with the irradiation strategy and especially the rotation angle of subsequent layers. He reported that the negligible case corresponds with a 67° increment between layers, which was the only case in which Young’s modulus was independent of the azimuth angle. On a side note, a further possibility for promoting isotropic material behaviour through a second irradiation of each layer, with a 90° rotation increment between the two irradiations, was reported by AlMangour et al. [57].

The breaking elongation was considerably higher for both cases with *Θ* = 90°, which increased by 28.5% (config. (a) to (b)) and 48.5% (config. (f) to (g)), respectively. These findings coincided with those of Meier and Haberland [18], who also reported fluctuations of the breaking elongation with a varying azimuth angle.

**Figure 5:**
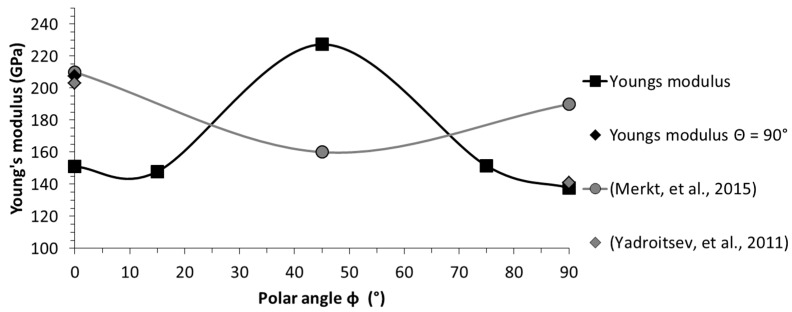
Orientation dependency of Young’s modulus; comparison with reported results [4,65].

**Figure 6:**
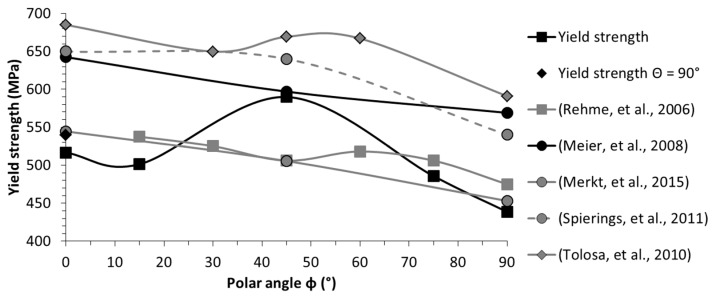
Orientation dependency of the yield strength; comparison with reported results [4,14,18,20,21].

**Figure 7:**
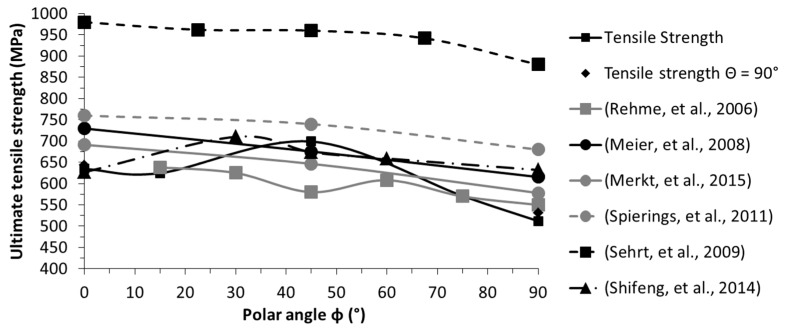
Orientation dependency of the ultimate tensile stress; comparison with reported results [4,14,18,19,20,66].

**Figure 8:**
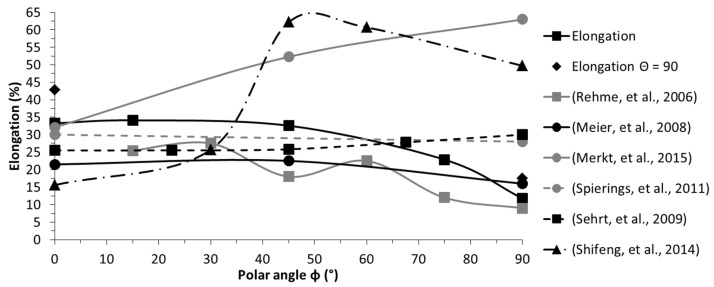
Orientation dependency of the elongation at fracture; comparison with reported results [4,14,18,19,20,66].

Nonetheless, the results of this investigation are in perfect agreement with the polar angle being the major directional dependency, influencing all tensile characteristics. The polar angle dependencies of each single characteristic are depicted in detail in Figure 5, Figure 6, Figure 7 and Figure 8 and compared with the results of related studies. Interestingly, the maxima for the Young’s modulus and the tensile strength were evident for *Φ* = 45°. On a side note, given this superimposition with the azimuth angle dependency, it can be anticipated that in the case at hand the combination of *Φ* = 45° and *Θ* = 90°, which was not investigated in the present study, would yield the highest results with the utilized manufacturing settings. Fluctuations in the strength (yield and ultimate tensile strength) and, especially, in the breaking elongation by alterations of the polar angle were frequently reported. However, alterations in the linear elastic behaviour have been investigated in far less detail and the few existing studies are not in agreement. In addition to the depicted results (Figure 5), Rehme and Emmelmann [20] stated that there is no evidence of dependency of the Young’s modulus on the polar angle. Based on the results of this work, however, there were remarkable deviations. The nature of these deviations has not been clarified holistically yet, and will be addressed in a future work.

The specific progress of the tendencies in the tensile strength values also differ noticeably between independent studies (Figure 6 and Figure 7). Considering the general formulation about the occurrence of the highest strength and breaking elongation in the parallel layer to loading orientation, it can be concluded that this is indeed correct in many instances, but only partially correct when considering the big picture, and far from being applicable as a general rule. The tensile strength, whether yield or ultimate strength, showed a general tendency to be higher for the parallel loading to layer case. However, the progression in between the extrema (i.e., parallel and perpendicular) did not follow a general rule. It differed greatly by chosen manufacturing settings and setup across these various studies, and it is also highly volatile in relation to individual material characteristics. For a conventional bulk base material, there is a direct correlation between the ultimate tensile strength and the surface hardness, and concluding one characteristic from the other is well-known, e.g., the German standard for conversion of hardness values to ultimate tensile strength for steel DIN EN ISO 18265:2014-2 [58]. When comparing the fluctuating results for the ultimate tensile strength (Table 6), ranging from 512 to 699 MPa, with the constant hardness results (Table 4), it can be concluded that this conversion is not constructive for selective laser-melted samples. A few similar findings were reported in the literature, which indicate that the correlation of the tensile strength and surface hardness is not given in AM: Mertens et al. [59] showed that through an optimized preheating temperature, the ultimate tensile strength of 1.2344 could be drastically increased; this increase, however, was coupled with a decrease in hardness. Abd-Elghany and Bourell [34] argued that this incongruity is reasoned to be due to the differing thermal environments, i.e., close to the free-surface and core.

Proceeding to the breaking elongation, this appeared to be by far the characteristic most volatile to alterations in orientation (Figure 8). In this instance, no clear tendency was evident, the range of reported results scattered greatly, and the progression behaviour appeared random at first glance, leading to the question of how this can be the case. Clearly, to answer this question holistically, more work needs to be done. For now, the major causes of these deviations are anticipated to be inherent residual stresses and incomplete fusion between scan tracks and layers. Both result in a weakening of the material in a predominant direction but, depending on where the defect occurs, the weakening varies in its predominant direction. Furthermore, these effects are greatly influenced by the laser power utilized and the ability to control the thermal environment, e.g., according to the range of available preheating temperatures, which alter microstructural development. On a side note, Wang et al. [60] and AlMangour et al. [61] reported that defects and pores in the as-built state can be overcome, to a great extent, by applying a subsequent hot isostatic pressing (HIP) treatment. Leuders et al. [62] have found that HIP increases the ductility of 1.4404, but due to the reduction in strength and its already good properties in the as-built state a post-heat treatment is not required in most cases. However, due to the inherent residual stresses, which can go far beyond the yield strength of the equivalent wrought material, heat treatments prior to removal from the substrate plate need to be carried out to avoid deformations and correlated accuracy issues [63]. On a side note, the residual stresses in SLM have a predominant direction and are generally higher in the direction of the scan track [64].

### 3.4. Comparison AM and Bulk Base Material

**Figure 9:**
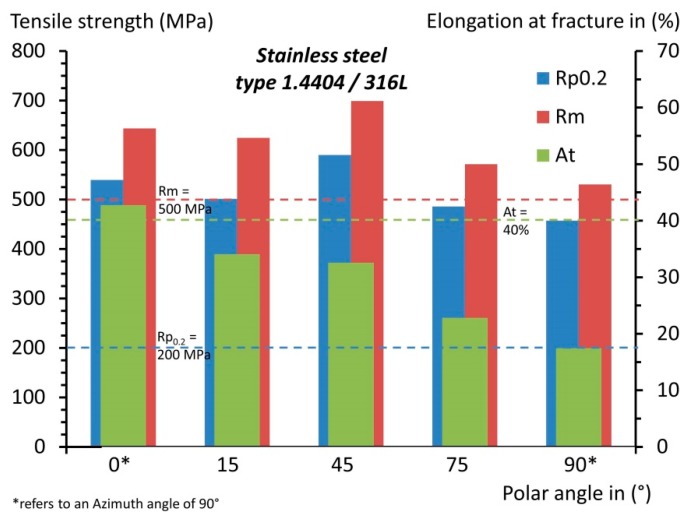
Comparison of additively manufactured and bulk base 1.4404 [67].

Additively manufactured material generally exhibits higher strength, coupled with reduced ductility (Figure 9). Yield and ultimate tensile strength were exceeded by far, yet the 40% minimum breaking elongation of bulk base 1.4404 is a criterion that was only achieved by one out of seven configurations. One other aspect that should be emphasised is the greatly reduced difference between the yield point and the ultimate tensile strength caused by AM [8]. In Table 7, several achieved ratios for 1.4404 are compared with its bulk base material. It should be noted that a similar finding was also reported for 1.4307 (US 304L) [34]. For safety reasons, it is beneficial if the difference between these measures is large. Thus, this peculiarity should be noted for components designed for fabrication using AM. It should be pointed out that this behaviour is not particularly negative, but simply something that needs to be considered. Among materials with high strength, this is very common, and AM also introduces this behaviour in materials that do not show this trait when fabricated with conventional procedures.

### 3.5. Comprehensive Analysis of the Directional Dependencies and Their Origin

Next, the inherent directional dependencies are investigated further and, to emphasise that their formation is greatly influenced by the chosen material, a direct comparison is made with a previous study on an aluminium-based die-cast alloy (AlSi10Mg) [16]. When comparing the polar angle dependencies of 1.4404 with the dependencies of AlSi10Mg, a clear influence of the material can be shown. AlSi10Mg exhibited the lowest tensile strength under *Φ* = 45°, which is precisely where 1.4404 had its peak strength. The explanation for this finding is based on microstructural development during AM fabrication. The microstructure of AlSi10Mg revealed clear traces of every single scan track, which inherits an inhomogeneous nature. The Al grain boundaries are surrounded with Si particle segregations, which mainly take place in the connection between scan tracks and layers, namely hatch overlaps and wetting areas (see Figure 1a) [68,69]. These segregations on the grain boundaries have a stabilization effect and prevent secondary grain-coarsening or growth. Furthermore, the Si-rich areas along the boundaries are comparably brittle, thus representing a weak spot for fracture. Given that the line scanning approach is most commonly coupled with a rotation angle in between subsequent layers, these brittle predetermined breaking points are far more emphasised between layers (in the z-direction), than is the case in either the x- or y-directions. Applying a tensile loading under 45° to the layers results in the maximum shear stress acting parallel to the layers, thus shearing the layers apart along these embrittled areas, which are rich in Si-segregations.

**Figure 10:**
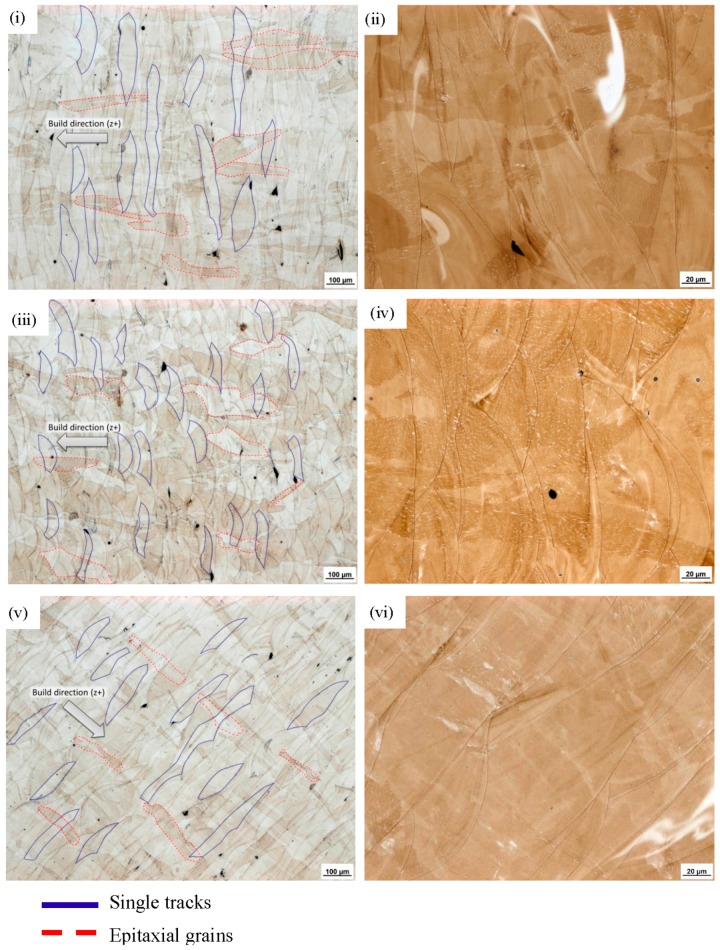
Microstructure of 1.4404, taken from the cross-section of the tensile samples of (**i**,**ii**) configuration (a); (**iii**,**iv**) configuration (b); and (**v**,**vi**) configuration (d). Left-hand column: lower resolution; right-hand column: higher resolution.

**Figure 11:**
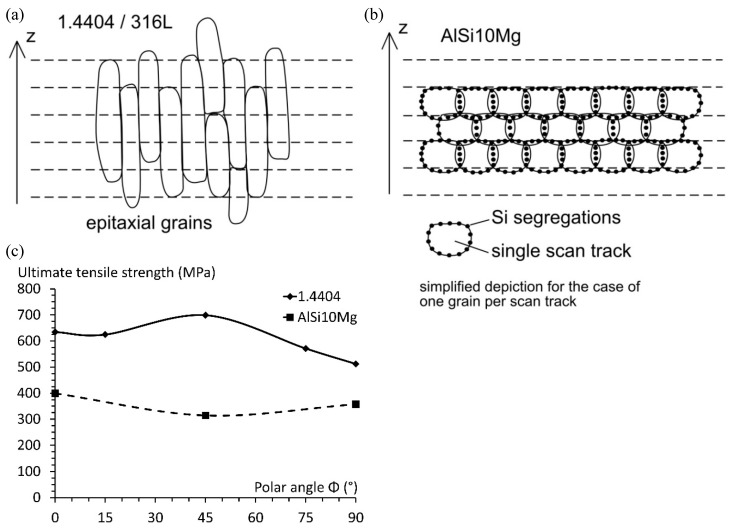
Comparison of the microstructural characteristics of (**a**) 1.4404 stainless steel and (**b**) AlSi10Mg, as well as their (**c**) strength dependency on the loading versus layer orientation [16].

Considering the microstructure of 1.4404, there are no obstacles for ongoing grain growth. Thus, subsequent heat inputs, due to the neighbouring scan track or subsequent layer being generated, alter the grains of the already solidified material [47]. This behaviour is commonly referred to as epitaxial grain growth, describing the tendency of needle-like grain growth towards the heat source [24,70]. Due to this tendency, the grains of 1.4404 grew through the individual layers (in the direction of the heat source), causing an interlocking of the individual layers (Figure 10). This interlock occurs in all directions, i.e., through layers and also through neighbouring scan tracks. Since the scan track direction is altered between layers, this interlocking mechanism can be seen best in the build-up direction. An exemplified depiction of these underlying mechanisms is depicted in Figure 11 and a direct comparison of the obtained polar angle dependencies is provided as well, which clearly points out the resulting opposing, material-dependent, progressions. Further examples of the material dependency on the development of directional anisotropies in AM are found in the literature. In the work of Sehrt [1], it was seen that the NiCr alloy (Hastelloy X) developed a more emphasised polar angle dependency than the stainless steel GP1. The results of Spierings et al. [71] on an AlMgSc alloy (Scalmalloy) suggested that, for this material, the polar angle dependency can be neglected entirely, since the reported deviations are below 3.4%. Given this, there cannot be a true generalized statement on the inherent anisotropic character of AM-fabricated components. At the least, differentiations on the underlying material have to be made.

Returning to the micro-sections (Figure 10), in these can also be found the answer for the deviations regarding the azimuth angle that were encountered. A closer look at the shape of the single scan tracks yielded the following. The cross-section of configuration (a) samples mainly revealed oblong scan tracks, thus indicating that the majority of the scan tracks were fabricated around a perpendicular orientation to the longitudinal axis. For configuration (b), the other case was evident: single bead cross-sections can be seen clearly, indicating that the single scan tracks were predominantly fabricated parallel to the specimen’s longitudinal axis. These findings yield the following conclusion: in configuration (a), the loading occurred predominantly perpendicular to the scan tracks; whereas in the case of configuration (b), the loading occurred predominantly parallel to the scan tracks, with the latter yielding the higher strength.

**Figure 12:**
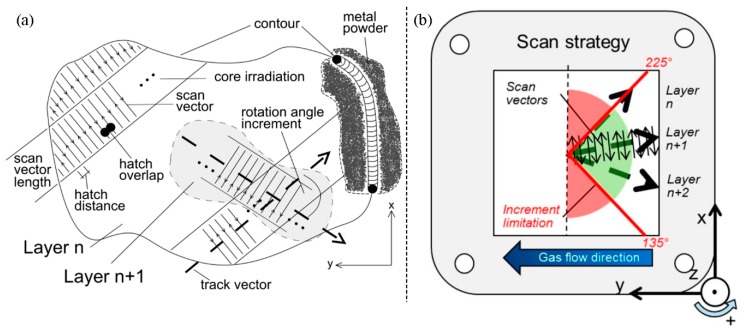
(**a**) Irradiation strategy and (**b**) limitation window; adapted from [8,72].

These differing stacking patterns are based on the applied irradiation pattern; more precisely, on the limitation window, which limits the admissible range of irradiation directions. Its main purpose is to exclude all scan directions that result in a laser irradiation movement towards the inert gas stream. The reason for this limitation is to prevent any interaction of emerging weld splashes and smoke with the ongoing irradiation [72]. These particles get transported out of the build chamber via the inert gas flow; hence, a laser movement in the direction of the inert gas stream is likely to cause unwanted interactions with these particles and can cause defects in the fabrication process due to a lack of highly focused power. However, on the other hand this precaution limits the range of possible rotation increments of subsequent layers and causes the occurrence of predominant directions. In this study, the allowed range of possible track vectors was 90°, which limits the possible track vector range to ±45°, with the track vectors always pointing in the negative y-direction (opposed to the inert gas stream), resulting in the lower increment border at 135° and the upper border at 225° (Figure 12), respectively. With the rotation increment set to 33° (Table 1), the sequence of track vectors and scan track vector angles, as outlined in Table 8, arises.

The findings show that in SLM the properties of the generated part are greatly affected by the applied scanning pattern, and a thorough prediction of the properties of additively manufactured components prior to fabrication is, consequently, a challenge. Since the SLM process represents a micro-welding process, in a well-controlled build chamber, the general correlation between the solidification of a scan track and its microstructure is similar to the weld microstructures. Therefore, the microstructural characteristics of a single scan track follow the rules of a rapid solidification starting from the melt pool boundary, i.e., the contact between the melt and the solid material [73]. It should be noted that the energy input, although the laser source operates during the core irradiation at a constant speed and irradiation intensity, is not constant. Trapp et al. [74] investigated intensively the fluctuations and alterations of the absorptivity in the powder-bed, and showed that the real absorption differs greatly from the known values from powder layer and liquid metal estimates. Hence, it can be concluded that, although the physics of welding applies, the changes and interactions between powder state, liquid state, and consolidated metal state render a thorough prediction more complicated, and that more work needs to be done for a full understanding. On a side note, for powder-bed based AM techniques the single scan tracks are two-dimensional only. When considering nozzle-fed AM technologies, the scan strategy becomes a three-dimensional construct, introducing further complexity in terms of the prediction of the properties [75].

## 4. Conclusions

In this study, the peculiarities of additively manufactured material were addressed using the example of stainless steel. It was shown that homogeneous structures can be fabricated, and preheating temperatures of up to 200 °C do not cause location-dependent alteration of the microstructure. The scan strategy was found to influence the material characteristics significantly and even simple precautions, such as limiting the irradiation pathways to avoid possible interactions between emerging particles with the laser beam, promote inherent directional dependencies. In addition, the general rule of higher strength occurring in a parallel layer to the loading direction, in comparison with the perpendicular layer to loading scenario, was proven accurate. However, the progression of the mechanical characteristics by altering the inclination between the loading and the layers differed, and was shown to be highly material-dependent. Stainless steel exhibited its peak strength and maximum Young’s modulus under a 45° offset between the layer and loading direction, whereas the aluminium–silicon alloy AlSi10Mg revealed the lowest strength in this instance. In regard to the breaking elongation, the tested specimen showed a noteworthy drop in ductility past an inclination offset of 45°. Considering the disparate tendencies found in related studies, it can be concluded that the orientation dependency of the ductility in AM is, to date, not fully understood and further in-depth investigations need to be undertaken. Moreover, future work is aimed at the modification and extension of classical welding theory to enable the prediction of additively manufactured components prior to fabrication.

## Figures and Tables

**Table 1:** Parameter sets utilized for irradiation in SLM for the processing of 1.4404; see description of the parameters and the setup in Figure 1.

Parameter Set	Scan Speed (mm/s)	Laser Power (W)	Hatch Distance (mm)	Rotation Angle Increment (°)	Energy Density (J/mm^3^)
Contour	400	100	0.09	-	92.6
Core	800	200	0.12	33	69.4
Final layer	400	300	0.1	-	250.0
Support	875	200	-	-	-
Common	Layer thickness of 30 μm				
	Mounting plate temperature of 200 °C				
	Nitrogen is employed as the inert gas				
	Contour is irradiated first, followed by the core, utilising the line scanning strategy with a scan vector length of 10 mm				
	Limitation window of 90°, respectively ±45° to the *y*-axis				

**Table 2:** Summary of positioning details for all considered configurations, grouping of individual manufacturing jobs and corresponding time per job.

Config.	Polar Angle *Φ; α*_XY_ (°)	Azimuth Angle *Θ*; *α*_X_ (°)	Total Runtime (h)
(a)	0	0	39.5
(b)	0	90	
(c)	15	0	86.5
(d)	45	0	
(e)	75	0	
(f)	90	0	
(g)	90	90	

Slight deviations from 0° to 90° angles were introduced for the azimuth angle of the in-plane oriented configurations ((a) and (b)) to improve the recoating process by ensuring that its blade does not abruptly hit the entire edge at once.

**Table 3:** Chemical composition of the specimens, all values in weight-percent.

Config.	Fe	C	Si	Mn	P	S	Cr	Ni	Mo	N
(a)–(b)	Bal	0.031	0.564	1.044	<0.005	0.007	16.837	11.691	2.371	-
(c)–(g)	Bal	0.0235	0.585	1.051	<0.005	<0.005	16.994	11.257	2.390	-
DIN EN 10088-3	Bal	<0.03	<1	<2	<0.045	<0.03	16.5–18.5	10–13	2–2.5	<0.1

**Table 4:** Surface hardness results per configuration obtained on the machined samples.

Config.	Vickers Hardness Core (HV30)	Standard Deviation Core (HV30)	Vickers Hardness Contour (HV30)	Standard Deviation Contour (HV30)
(a)	226.7	6.1	242.8	18.4
(b)	234.4	8.0	245.0	12.2
(c)	229.9	5.6	243.3	26.9
(d)	227.8	7.2	235.5	11.0
(e)	230.4	6.2	240.6	14.3
(f)	223.6	7.9	236.6	13.7
(g)	229.2	8.3	235.2	9.3

**Table 5:** Hardness results for 1.4404, comparison between literature and supplier specification; relative densities (≥99%).

Reference	Vickers Hardness	Machine	Max. Laser Power [W]
This work	223–245 HV30	SLM 280HL	400
Cherry, et al. [10]	220–225 HV	Renishaw AM250	200
Tolosa, et al. [21]	215–255 HV mean of 235 HV	SLM 250 Realizer	-
Kruth, et al. [50]	220–250 HV0.1	-	-
Montani, et al. [51]	245 HV	Prototype, not further specified	1000
Sheet metal, typical value [52,53,54]	~220 HV(212–217 HB)	-	-

**Table 6:** Averaged results for the tensile properties of 1.4404.

Config.	Young’s Modulus *E* (GPa)		Yield Strength *R*_p0.2_ (MPa)		Ultimate Tensile Strength *R*_m_ (MPa)		Elongation at Failure *A*_t_ (%)		Poisson’s Ratio *ν* (-)	
	Average	STDEV	Average	STDEV	Average	STDEV	Average	STDEV	Average	STDEV
(a)	151.01	25.56	516.51	7.16	634.43	7.39	33.24	0.57	0.444	0.031
(b)	207.57	24.22	539.47	3.29	643.67	3.25	42.74	0.82	0.155	0.014
(c)	147.87	23.59	501.32	7.70	624.65	4.36	34.09	1.12	0.479	0.058
(d)	227.35	25.12	589.89	11.86	698.98	23.65	32.56	10.17	0.203	0.024
(e)	151.43	18.80	485.65	11.93	571.23	18.63	22.84	7.27	0.558	0.020
(f)	137.78	14.25	438.60	9.69	511.99	17.95	11.76	5.38	0.453	0.005
(g)	137.83	16.25	457.21	17.29	530.22	8.09	17.46	4.42	0.170	0.085

**Table 7:** Ratio between the yield point and the ultimate tensile strength for 1.4404.

Reference	Configurations	Range *R*_e_/*R*_m_ (-)	Averaged * Ratio *R*_e_/*R*_m_ (-)
this work	7	0.8026–0.8623	0.8383
Meier and Haberland [18]	5	0.8621–0.9261	0.8889
Merkt [4]	3	0.7819–0.7877	0.7844
Rehme and Emmelmann [20]	150	0.8400–0.8877 **	0.8597 **
Riemer, et al. [47]	1	0.8177	-
	heat treated 2 h, 650 °C	0.7445	-
Spierings, et al. [14]	3	0.7941–0.8648	0.8380
Tolosa, et al. [21]	15	0.9163–0.9967	0.9565
bulk base 1.4404 [67]	-	0.4	-

* Investigated configurations are valued equally, sample sizes neglected; ** only *Θ* = 0° and *Φ* = 0°–90° considered.

**Table 8:** Exemplary calculation of the direction of subsequent irradiation tracks.

Layer	Track Vector Angle	Scan Vector Angle
1	(bottom increment limitation border) = 135°	(track vector angle) ± 90° = 45°; 225°
2	(previous track vector angle) + (rotation angle increment) = 135° + 33° = 168°	(track vector angle) ± 90° = 78°; 258°
3	(previous track vector angle) + (rotation angle increment) = 168° + 33° = 201°	(track vector angle) ± 90° = 111°; 291°
4	would be outside the limitation window!, thus: (previous track vector angle) + (rotation angle increment)—(top Increment limitation border) + (bottom Increment limitation border) = 201° + 33° − 225° + 135° = 144°	(track vector angle) ± 90° = 54°; 234°
5	(previous track vector angle) + (rotation angle increment) = 144° + 33° = 177°	(track vector angle) ± 90° = 87°; 267°
